# Endogenous Origins of HIV-1 G-to-A Hypermutation and Restriction in the Nonpermissive T Cell Line CEM2n

**DOI:** 10.1371/journal.ppat.1002800

**Published:** 2012-07-12

**Authors:** Eric W. Refsland, Judd F. Hultquist, Reuben S. Harris

**Affiliations:** 1 Department of Biochemistry, Molecular Biology, and Biophysics, University of Minnesota, Minneapolis, Minnesota, United States of America; 2 Institute for Molecular Virology, University of Minnesota, Minneapolis, Minnesota, United States of America; 3 Center for Genome Engineering, University of Minnesota, Minneapolis, Minnesota, United States of America; 4 Masonic Cancer Center, University of Minnesota, Minneapolis, Minnesota, United States of America; 5 Department of Molecular, Cellular, Developmental Biology and Genetics, University of Minnesota, Minneapolis, Minnesota, United States of America,; Duke University Medical Center, United States of America

## Abstract

The DNA deaminase APOBEC3G converts cytosines to uracils in retroviral cDNA, which are immortalized as genomic strand G-to-A hypermutations by reverse transcription. A single round of APOBEC3G-dependent mutagenesis can be catastrophic, but evidence suggests that sublethal levels contribute to viral genetic diversity and the associated problems of drug resistance and immune escape. APOBEC3G exhibits an intrinsic preference for the second cytosine in a 5′CC dinucleotide motif leading to 5′GG-to-AG mutations. However, an additional hypermutation signature is commonly observed in proviral sequences from HIV-1 infected patients, 5′GA-to-AA, and it has been attributed controversially to one or more of the six other APOBEC3 deaminases. An unambiguous resolution of this problem has been difficult to achieve, in part due to dominant effects of protein over-expression. Here, we employ gene targeting to dissect the endogenous APOBEC3 contribution to Vif-deficient HIV-1 restriction and hypermutation in a nonpermissive T cell line CEM2n. We report that *APOBEC3G*-null cells, as predicted from previous studies, lose the capacity to inflict 5′GG-to-AG mutations. In contrast, *APOBEC3F*-null cells produced viruses with near-normal mutational patterns. Systematic knockdown of other *APOBEC3* genes in an *APOBEC3F*-null background revealed a significant contribution from APOBEC3D in promoting 5′GA-to-AA hypermutations. Furthermore, Vif-deficient HIV-1 restriction was strong in parental CEM2n and *APOBEC3D*-knockdown cells, partially alleviated in *APOBEC3G*- or *APOBEC3F*-null cells, further alleviated in *APOBEC3F*-null/*APOBEC3D*-knockdown cells, and alleviated to the greatest extent in *APOBEC3F*-null/*APOBEC3G*-knockdown cells revealing clear redundancy in the HIV-1 restriction mechanism. We conclude that endogenous levels of APOBEC3D, APOBEC3F, and APOBEC3G combine to restrict Vif-deficient HIV-1 and cause the hallmark dinucleotide hypermutation patterns in CEM2n. Primary T lymphocytes express a similar set of *APOBEC3* genes suggesting that the same repertoire may be important *in vivo*.

## Author Summary

HIV uses its accessory protein Vif to shield itself from lethal mutagenesis by cellular APOBEC3 proteins. The APOBEC3-Vif interaction is therefore a promising target for drug development, and a clear definition of all proteins involved on both sides of this critical host-pathogen conflict is crucial. However, apart from APOBEC3G, conflicting over-expression studies have been published on the six other human APOBEC3s, some supporting and some refuting each protein's involvement in HIV restriction. Here, we use a combination of gene targeting and RNA interference to define the endogenous sources of Vif-deficient HIV restriction and G-to-A hypermutation in a nonpermissive T cell line. As anticipated, APOBEC3G contributed partially to restriction and proved to be the source of hypermutations at 5′GG dinucleotides. However, a major and unexpected role for APOBEC3D became evident in the *APOBEC3F*-null background, with both proteins combining to suppress viral infectivity and form hypermutations at 5′GA dinucleotides. We conclude that endogenous levels of APOBEC3D, APOBEC3F, and APOBEC3G act redundantly to restrict Vif-deficient HIV and produce the two hallmark dinucleotide hypermutation patterns observed in patient-derived viral sequences. Therapeutic strategies that unleash this full ‘swarm’ of restrictive APOBEC3 proteins are likely to be more effective than those that focus on enabling a single enzyme.

## Introduction

Human cells can express up to seven APOBEC3 (A3) proteins: A3A, A3B, A3C, A3D, A3F, A3G, and A3H [1], [2]. A3G is the archetypal restriction factor, capable of restricting Vif-deficient HIV-1 (hereafter HIV) by packaging into viral cores and then suppressing reverse transcription and deaminating viral cDNA cytosines to uracils (C-to-U) (reviewed by [3], [4]). The hallmark of A3G activity is viral genomic plus-strand G-to-A mutations within 5′GG-to-AG dinucleotide motifs, which reflects its minus-strand 5′CC-to-CU preference [5], [6]. However, HIV single-cycle and spreading infection experiments have yet to provide an overall consensus to explain the additional 5′GA-to-AA dinucleotide bias that is also commonly found in patient-derived viral sequences [7], [8], [9], [10], [11]. In fact, over-expression studies have implicated all six of the other A3 proteins in generating this mutation pattern, with multiple studies for and against each enzyme (reviewed by [3]). For example, despite several early studies strongly implicating A3F as a major source of the 5′GA-to-AA mutations ([12], [13], [14], [15] and nearly twenty more thereafter), two recent papers have questioned its relevance to HIV restriction and hypermutation [16], [17]. The data are even murkier and more conflicting for the other five human APOBEC3 proteins (reviewed by [3]; summarized in Discussion).

Three major problems have made it difficult to address which endogenous A3 proteins cause HIV restriction and hypermutation. First, most prior studies have relied on transient or stable over-expression of a single A3 coupled to assays for viral infectivity and hypermutation. Although powerful for answering some questions, over-expression of a dominant and active DNA deaminase may overwhelm regulatory mechanisms and adversely affect the cell, result in nonspecific packaging into the virus, and create sequence artifacts by gratuitous deamination of non-productive viral replication intermediates. Most over-expression approaches are complicated further by being done in HEK293 (kidney) or HeLa (cervical) cells, which may not recapitulate as many aspects of restriction and/or viral replication as CD4+ T cell lines. Even best attempts to stably express physiological levels of APOBEC3 proteins in permissive CD4+ T cell lines are imperfect, because each A3 is expressed without other family members (*i.e.*, out of normal endogenous context) and the expression of each protein is driven by heterologous promoters with foreign 5′ and 3′ untranslated sequences that are unlikely to be responsive to cellular signaling pathways triggered by viral infection and/or interferon signaling [16], [18], [19], [20], [21], [22], [23]. Second, the seven human *A3* genes share high levels of nucleotide identity, which has hindered the development of gene-specific quantitative real-time (Q)-PCR assays and knockdown reagents. This problem is in the midst of being overcome with the development of robust Q-PCR assays [1], [2] and the creation of gene-specific knockdown constructs (this study and [24], [25], [26]). Finally, the field has yet to benefit from a robust genetic system, because HIV does not replicate in mouse models and the vast majority of human somatic cell lines are polyploid and/or difficult to engineer.

In this study, we use gene targeting and knockdown experiments to systematically interrogate the impact of the endogenous *A3* repertoire on Vif-deficient HIV replication in the near-diploid T cell line CEM2n. Null clones demonstrated that A3G is solely responsible for HIV 5′GG-to-AG hypermutations. *A3F*-null clones inflicted near-normal hypermutation patterns in Vif-deficient HIV, demonstrating that this enzyme alone is not responsible for 5′GA-to-AA hypermutations. Systematic depletion of other expressed *A3* mRNAs in the *A3F*-null background revealed an unanticipated, major role for A3D in generating 5′GA-to-AA hypermutations and restricting Vif-deficient HIV replication. We conclude that endogenous A3D and A3F combine to generate hallmark 5′GA-to-AA hypermutations and A3G generates the 5′GG-to-AG hypermutations. All three enzymes work together to suppress Vif-deficient HIV replication and generate the classical non-permissive phenotype.

## Results

### A New Model System for T Cell Genetics

**Figure 1:**
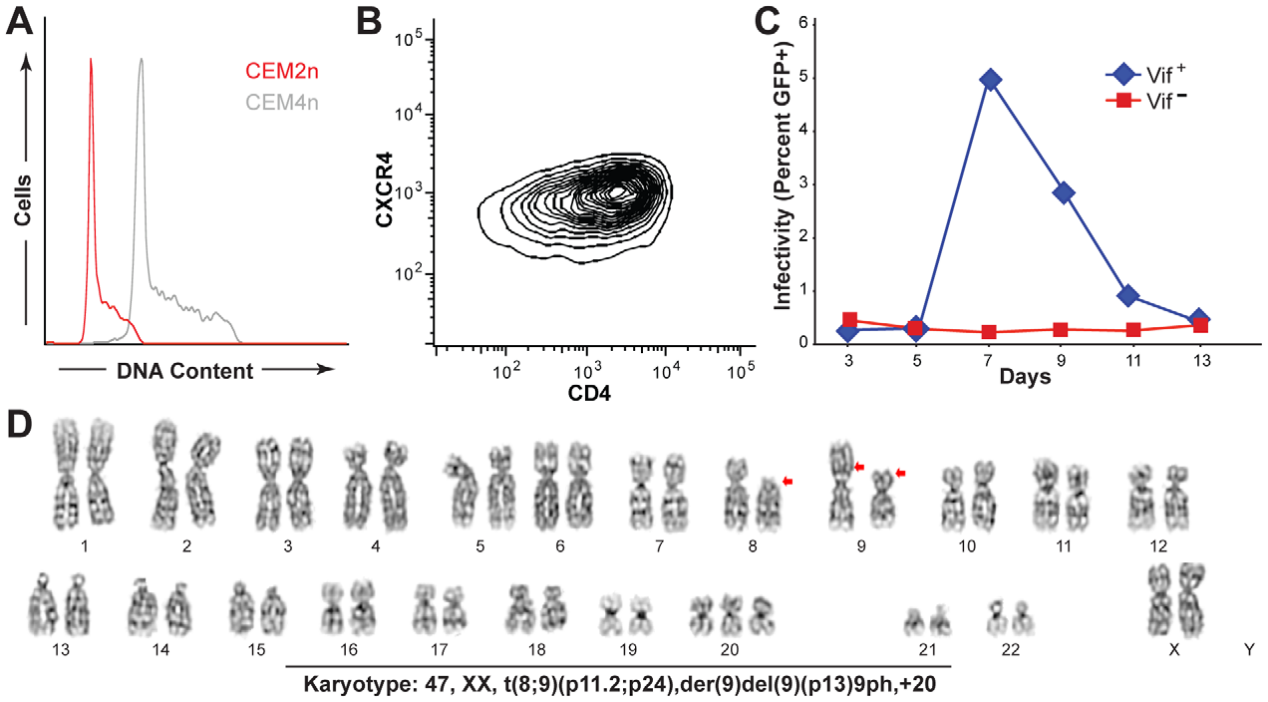
CEM2n is a near-diploid, non-permissive T cell line. (A) Flow cytometric analysis of fixed CEM2n (red) and CEM4n (gray) cells stained with propidium iodide. (B) Contour plot of CD4 and CXCR4 levels in CEM2n. (C) HIV spreading infection profiles in CEM2n as monitored by periodic infection of an LTR-GFP reporter cell line, CEM-GFP. (D) Giemsa-banding karyotype of a representative CEM2n metaphase spread. Red arrows indicate typical lesions in lymphoblastic leukemia.

The human T cell line CEM was originally isolated from a 3 year-old female with acute leukemia [27]. It is a model T cell system for HIV research that, along with its derivative CEM-SS, enabled the identification of A3G as a dominant HIV restriction factor [28]. However, our CEM laboratory stock proved sub-ideal for gene targeting because it tested near tetraploid (Figure S1). We noticed however that the original cytogenetic analyses of CEM had 47 chromosomes and a range of 41–95 [27]. We therefore obtained an early stock of CEM (CCRF-CEM), generated subclones by limiting dilution, and measured DNA content by flow cytometry. In comparison to our original CEM line, several of these subclones had half the DNA content and were most likely diploid (Figure 1A).

One representative subclone, hereafter called CEM2n, was selected for further characterization. Flow cytometry showed that CEM2n expresses high levels of CD4 and the HIV co-receptor CXCR4 (Figure 1B). CEM2n still manifests the classic non-permissive phenotype by supporting wildtype HIV replication while restricting Vif-deficient HIV, similar to our tetraploid non-permissive line CEM (Figure 1C), as described originally [28], [29]. As anticipated by this phenotype, Q-PCR demonstrated expression of multiple *A3* mRNAs (Figure S2 & below). Finally, karyotype analysis showed that CEM2n is near-diploid, with a total of 47 chromosomes, including three copies of chromosome 20 and a common T cell leukemia reciprocal translocation (Figure 1D). These characteristics indicated that CEM2n would be an appropriate model system to delineate the endogenous A3s involved in HIV restriction.

### Targeted Deletion of *APOBEC3G* in CEM2n

**Figure 2:**
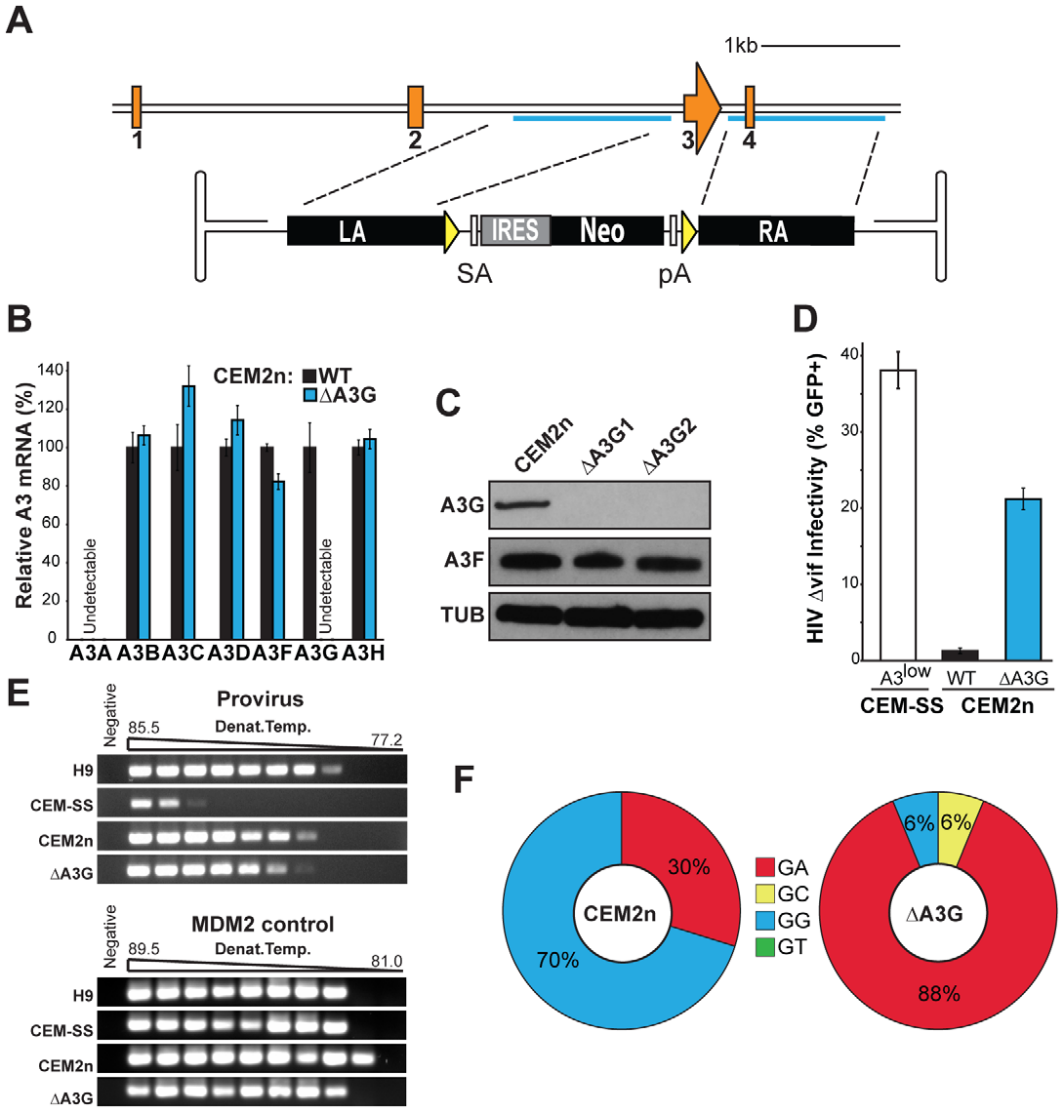
Construction and characterization of *A3G*-Null CEM2n cells. (A) *A3G* exon 3 targeting strategy. LA, left homology arm; SA, splice acceptor; IRES, internal ribosomal entry site; Neo, G418 resistance gene; pA, poly adenylation signal; RA, right homology arm; yellow triangles, *loxP* sites. (B) *A3* mRNA expression profiles of the indicated cells relative to parental CEM2n (mean and s.d. shown for triplicate experiments). (C) Immunoblots of A3G, A3F, and tubulin (TUB) in the indicated cells. (D) Infectivity of Vif-deficient HIV produced using the indicated cell lines following a single replicative cycle (mean and s.d. shown for p24-normalized triplicate experiments). (E) 3D-PCR profiles of HIV *gag-pol* and cellular *MDM2* targets within genomic DNA of infected CEM-GFP reporter cells. (F) HIV G-to-A mutation profiles of proviruses originating in the indicated cell types. The mutation frequency at each dinucleotide is illustrated as a pie chart wedge (n≥15 kb per condition).

Over 100 reports support a role for A3G in Vif-deficient HIV restriction (reviewed by [3]). A3G shows a strong bias for 5′GG-to-AG hypermutation, but it also has a secondary preference for 5′GA-to-AA invoking the formal possibility that it alone could be responsible for both dinucleotide signatures (*e.g.*, [5], [6]). To address this possibility, we used two rounds of rAAV-mediated gene targeting to generate *A3G*-null derivatives. The *A3G* targeting construct replaces exon 3, which encodes the N-terminal zinc-coordinating deaminase domain, with a promoterless drug resistance cassette (Figure 2A). A correctly targeted *A3G* gene is expected to be null because transcripts originating at the *A3G* promoter will splice to an acceptor sequence within the 5′ end of the cassette and then terminate with a polyA sequence at the 3′ end of the cassette (*i.e.*, the C-terminal two-thirds of the mRNA and protein should never be expressed). CEM2n was transduced with rAAV-A3G::Neo and drug resistant clones were selected with G418. PCR showed that 6/103 (5.8%) clones were targeted (Figure S3 & Table 1).

**Table 1:** Gene targeting statistics in CEM2n.

Gene	Allele 1	Allele 2	Allele 1 retargeted	Total clones analyzed	Overall targeting frequency (%)
*APOBEC3G*	6	2	4	189	6.3
*APOBEC3F*	7	3	2	170	7.1
**Totals:**	24			359	6.7

To delete the remaining *A3G* allele, the drug resistance cassette was removed by transducing a representative clone with a Cre expressing adenovirus, and then subclones with a *loxP*-to-*loxP* recombination event were identified by PCR screening (Figure S3). Next, the original rAAV-A3G::Neo construct was used for a second round of gene targeting. 2/86 drug resistant clones were null and 4/86 were retargeted, yielding a second round targeting frequency of 7.0% (Table 1). The *A3G*-null clones had undetectable levels of *A3G* mRNA and protein and, importantly, the mRNA levels of all of the flanking *A3* genes and the A3F protein levels were largely unperturbed (Figure 2B & C). The parental CEM2n line and its *A3G*-null derivatives had similar morphologies and growth rates.

To explore the functional consequences of deleting the endogenous *A3G* gene, we performed single-cycle infectivity assays with VSV-G pseudotyped Vif-deficient HIV_IIIB_. After one full round of replication, new viruses produced from *A3G*-null cells were used to infect CEM-GFP reporter cells and GFP fluorescence was measured 2 days later by flow cytometry to quantify infectivity. In comparison to the fully non-permissive parental line CEM2n, the *A3G*-null derivative lines produced viruses with approximately 10-fold improved infectivity (Figure 2D). However, these viruses were still 2-fold less infectious than Vif-deficient HIV produced in parallel using the related permissive T cell line, CEM-SS. We note that although CEM-SS is commonly accepted as permissive for Vif-deficient HIV replication, our recent work revealed that it expresses multiple A3s including low levels of A3G [2]. Thus, it is quite possible that a fully null derivative of CEM2n could produce even higher levels of infectious Vif-deficient HIV (further supported by experiments described below). Regardless, these data demonstrate that endogenous A3G is not the only factor contributing to Vif-deficient HIV restriction in CEM2n.

To gauge the gross level of G-to-A hypermutation in proviral DNA embedded in the genomes of the CEM-GFP reporter cells used above, we performed a series of differential DNA denaturation (3D-PCR) experiments [20], [30]. A 511 bp region of the HIV *gag-pol* gene was amplified over a range of PCR denaturation temperatures from 77.2 to 85.5°C and subjected to gel electrophoresis. As anticipated, Vif-deficient HIV proviruses derived from non-permissive T cell lines H9 and CEM2n yielded PCR products at low denaturation temperatures, down to 78.4 and 79.4°C, respectively, indicative of high levels of G-to-A hypermutation (Figure 2E). Vif-deficient HIV proviruses derived from CEM-SS only amplified at high denaturation temperatures, also as expected. In contrast, Vif-deficient proviruses derived from *A3G*-null cells yielded PCR amplicons at temperatures as low as 80.4°C, suggesting major levels of residual hypermutation but lower than those inflicted by the full A3 repertoire in CEM2n.

To examine mutational spectra, high temperature (unbiased) PCR amplicons were cloned and sequenced. As expected, Vif-deficient proviruses derived from the parental cell line CEM2n harbored extensive G-to-A hypermutations in two distinct dinucleotide contexts: 70% 5′GG and 30% 5′GA. This contrasted starkly to proviral sequences derived from *A3G*-null cells, in which the 5′GG-to-AG hypermutations nearly disappear (6%) (Figure 2F, Table 2, Figure S4, and Table S1). These results establish A3G as the major source of 5′GG-to-AG hypermutations, consistent with prior over-expression studies indicating that A3G is the only DNA deaminase that prefers minus strand 5′CC target sites (*e.g.*, [5], [6], [31], [32]. These mutation spectra also implicate at least one other DNA cytosine deaminase in restricting HIV and generating 5′GA-to-AA hypermutations.

**Table 2:** Mutation summary.

Cell line	Expt. no.	Number of 511 bp *gag-pol* amplicons	Total kb sequenced	Total GG-to-AG mutations	Total GA-to-AA mutations	Total GY-to-AY mutations	Total other mutations
CEM2n	1	30	15.3	40	17	0	8
CEM2n	2	30	15.3	21	30	0	3
ΔG	1	30	15.3	3	42	3	2
ΔF	2	30	15.3	18	21	0	6
ΔF shNS	3	30	15.3	31	30	1	5
ΔF shA3B	3	30	15.3	24	39	2	10
ΔF shA3C	3	30	15.3	10	7	1	9
ΔF shA3D	3	30	15.3	81	3	3	9
ΔF shA3G	3	30	15.3	4	4	0	19
ΔF shA3H	3	30	15.3	17	18	1	6

### Targeted Deletion of *APOBEC3F* in CEM2n

Q-PCR revealed that CEM2n cells express six of seven *A3* genes, *A3B*, *A3C*, *A3D*, *A3F*, *A3G*, and *A3H*, all of which have been implicated in catalyzing the 5′GA-to-AA hypermutation patterns (Figure S2, Discussion, and examples [12], [13], [14], [15]). A3F was our top candidate because (i) *A3F* expression tracks with *A3G* in non-permissive T cell lines, primary lymphocytes, and secondary immune tissues [2], [13], [20], (ii) A3F is encapsidated into budding viruses and restricts Vif-deficient HIV when over-expressed in permissive T cell lines [18], (iii) Vif targets A3F for degradation [14], [15], [18], (iv) A3F restriction capability and Vif counteraction activity is conserved with rhesus macaque A3F and SIV Vif [20], [33], and (v) Vif-deficient HIV isolates that regain the capacity to replicate on A3F expressing cells invariably restore Vif function [18]. However, despite this strong evidence favoring a role for A3F in HIV restriction and hypermutation, recent studies have questioned its importance [16], [17].

**Figure 3:**
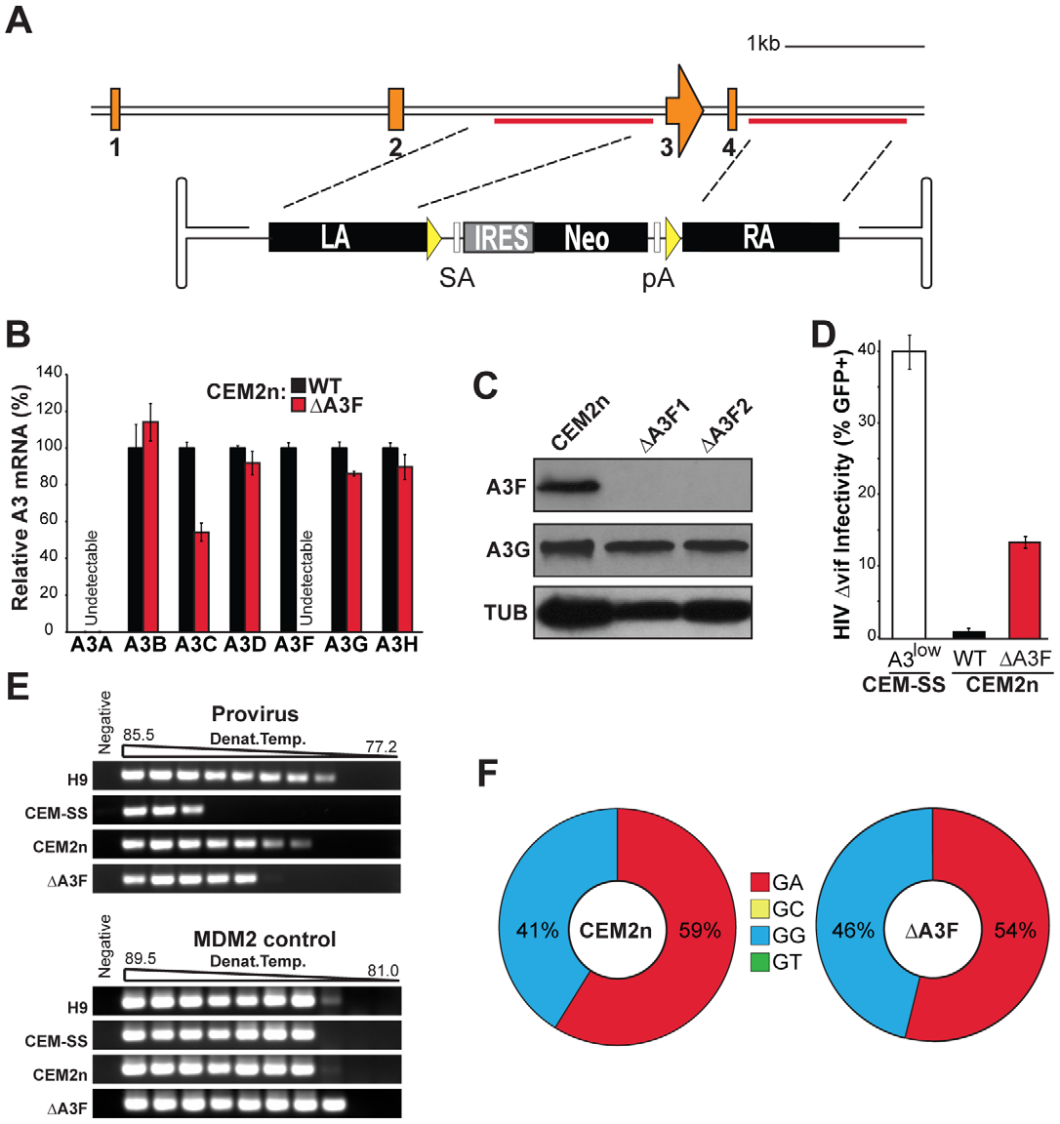
Construction and characterization of *A3F*-Null CEM2n cells. (A) *A3F* exon 3–4 targeting strategy. LA, left homology arm; SA, splice acceptor; IRES, internal ribosomal entry site; Neo, G418 resistance gene; pA, poly adenylation signal; RA, right homology arm; yellow triangles, *loxP* sites. (B) *A3* mRNA expression profiles of the indicated cells relative to parental CEM2n (mean and s.d. shown for triplicate experiments). (C) Immunoblots of A3F, A3G, and tubulin (TUB) in the indicated cells. (D) Infectivity of Vif-deficient HIV produced using the indicated cell lines following a single replicative cycle (mean and s.d. shown for p24-normalized triplicate experiments). (E) 3D-PCR profiles of HIV *gag-pol* and cellular *MDM2* targets within genomic DNA of infected CEM-GFP reporter cells. (F) HIV G-to-A mutation profiles of proviruses originating in the indicated cell types. The mutation frequency at each dinucleotide is illustrated as a pie chart wedge (n≥15 kb per condition).

To determine the involvement of endogenous A3F in Vif-deficient HIV restriction and hypermutation, we generated *A3F*-null cell lines using two rounds of rAAV-mediated gene targeting as described above (Figure 3A and Figure S5). *A3F*-null clones showed no detectable mRNA or protein, and the mRNA levels of all of the flanking *A3* genes and the A3G protein levels were largely unaffected (Figure 3B & C). Similar to the loss of *A3G*, the deletion of *A3F* resulted in cells semi-permissive for Vif-deficient HIV (Figure 3D) as well as in a modest decrease in the overall level of mutation as gauged by 3D-PCR (Figure 3E). However, we were surprised to find that the hypermutation spectrum of Vif-deficient HIV produced in *A3F*-null cells was indistinguishable from that of the same virus produced in the CEM2n parent, retaining a large percentage of 5′GA-to-AA mutations (Figure 3F, Table 2, Figure S4, and Table S1). Thus, the infectivity data showed that endogenous A3F does contribute to Vif-deficient HIV restriction, but the hypermutation data clearly implicate at least one other endogenous 5′TC deaminating A3.

### APOBEC3D and APOBEC3F Combine to Cause 5′GA-to-AA Hypermutations

**Figure 4:**
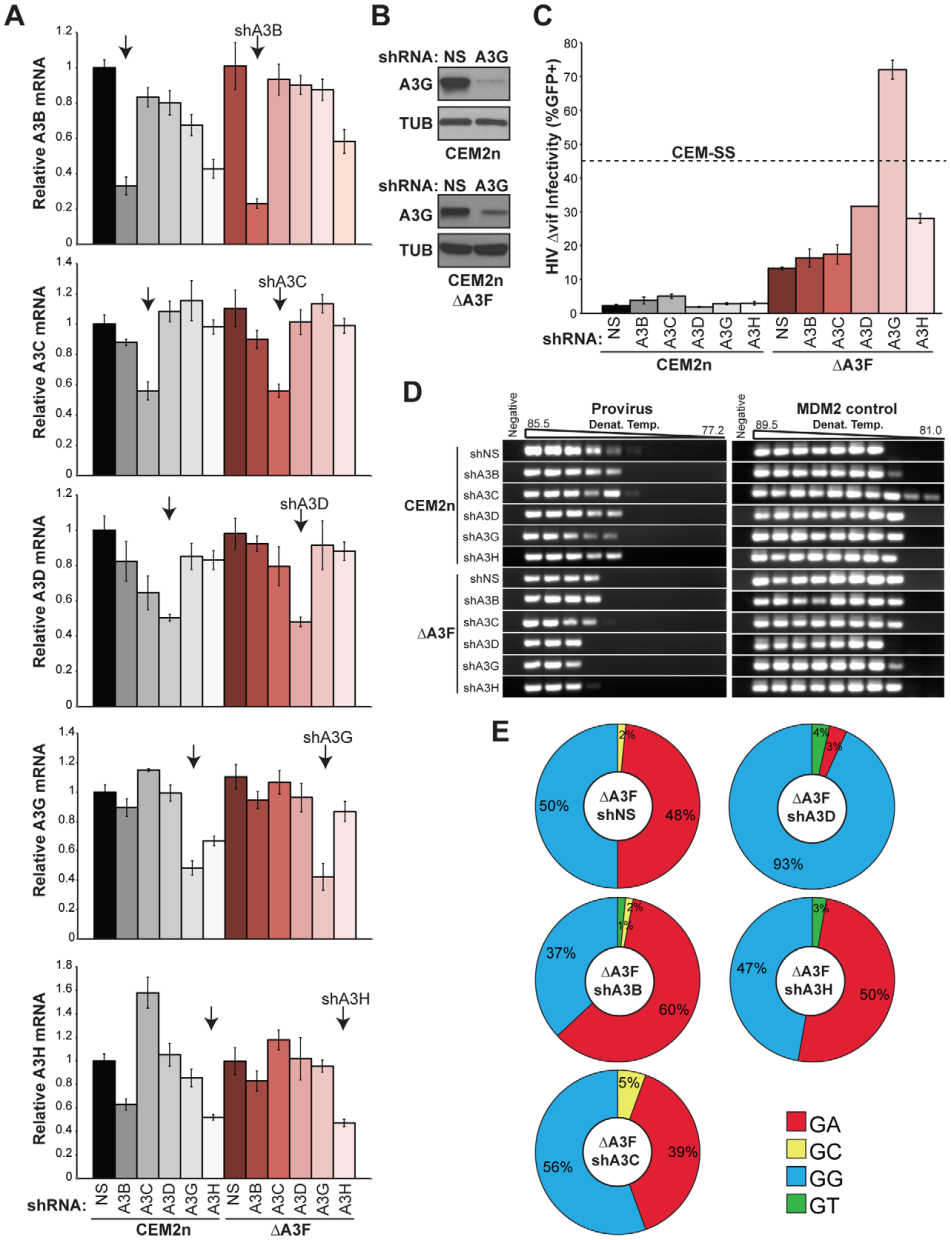
Construction and characterization of *A3F*-Null/*A3*-Knockdown CEM2n cells. (A) Levels of each indicated *A3* mRNA in CEM2n or *A3F*-null cells transduced with shNS, shA3B, shA3C, shA3D, shA3G, or shA3H constructs (mean and s.d. shown for triplicate experiments). (B) Immunoblots of A3G and tubulin (TUB) in CEM2n or *A3F*-null cells stably transduced with the indicated shRNA-expressing lentivirus. (C) Infectivity of Vif-deficient HIV produced using the indicated transduced cell pool and reported using the CEM-GFP system (mean and s.d. shown for p24-normalized triplicate experiments; in some instances, the error is nearly indistinguishable from the histogram bar outline). (D) 3D-PCR profiles of HIV *gag-pol* and cellular *MDM2* targets within genomic DNA of infected CEM-GFP reporter cells. (E) HIV G-to-A mutation profiles of proviruses originating in the indicated cell types. The mutation frequency at each dinucleotide is illustrated as a pie chart wedge (n≥15 kb per condition). Pie charts were generated for those conditions with ≥1 mutation per kb analyzed. Mutation numbers for all conditions can be found in Table 2 and Table S1.

To identify the remaining source of the 5′GA-to-AA hypermutations, we developed a panel of short hairpin (sh)RNA reagents to systematically knockdown the expression of *A3B*, *A3C*, *A3D*, *A3G*, and *A3H* in the *A3F*-null background (Figure 4A). Knockdown efficiencies ranged from 50–80% and were specific to each intended *A3* mRNA target. These efficiencies may be even higher at the protein level due to the known effects of shRNA in triggering mRNA cleavage/degradation and in suppressing translation [34], but this could only be confirmed for A3G due a lack of specific antibodies for the other A3s (Figure 4B). The parental CEM2n and *A3F*-null lines were transduced with non-silencing (shNS) and each of the aforementioned shA3 constructs. Pools of shRNA expressing cells were selected with puromycin (co-expressed from the same transducing virus), subjected to knockdown verification by Q-PCR, and infected as above with VSV-G pseudotyped Vif-deficient HIV to determine infectivity levels and hypermutation patterns.

Each individual *A3* knockdown had little impact on the infectivity or the 3D-PCR profile of Vif-deficient HIV produced in the CEM2n parental line (Figure 4C, 4D). *A3F*-null cells expressing the non-silencing shRNA yielded a significant 7-fold increase in the infectivity of Vif-deficient HIV over parental CEM2n as above (Figure 4C). Similarly, *A3F*-null cells transduced with shA3B or shA3C knockdown constructs produced Vif-deficient viruses with 8- and 9-fold infectivity increases, indicating no further contribution to restriction from these A3 proteins (Figure 4C). However, in contrast, *A3F*-null cells transduced with shA3D, shA3G, and shA3H constructs yielded Vif-deficient viruses that were 16-, 36-, and 14-fold more infectious, respectively (Figure 4C). Remarkably, the *A3F*-null/*A3G*-knockdown cells produced higher levels of infectious Vif-deficient HIV than CEM-SS cells (Figure 4C).

Proviral DNA sequencing of Vif-deficient viruses produced in *A3F*-null cells in combination with non-specific shRNA (shNS), shA3B, shA3C, or shA3H showed no significant alteration in the fraction of hypermutations that occurred within 5′GA or 5′GG dinucleotides (Figure 4E, Table 2, and Table S1). Over 15 G-to-A mutations were found for all experimental conditions, with one exception being viral DNA originally produced in *A3F*-null/*A3G* knockdown cells, which yielded only 8 mutations (4 GG-to-AG and 4 GA-to-AA). In contrast, *A3F*-null/*A3D* knockdown cells produced almost no hypermutations in the 5′GA-to-AA dinucleotide context: 3% 5′GA, 93% 5′GG, and 4% other (Figure 4E, Table 2, Figure S4, and Table S1). This major contribution from A3D was only observed in the absence of A3F and it was rather surprising because most other reports have ascribed modest or no antiretroviral activity to this enzyme ([12], [19], [35], [36]; reviewed recently [3]). We conclude that, in CEM2n cells, endogenous A3D and A3F combine to restrict Vif-deficient HIV (with A3G) and, importantly, work together (mostly without A3G) to inflict 5′GA-to-AA hypermutations.

### Knockout and Knockdown Subclone Analyses

**Figure 5:**
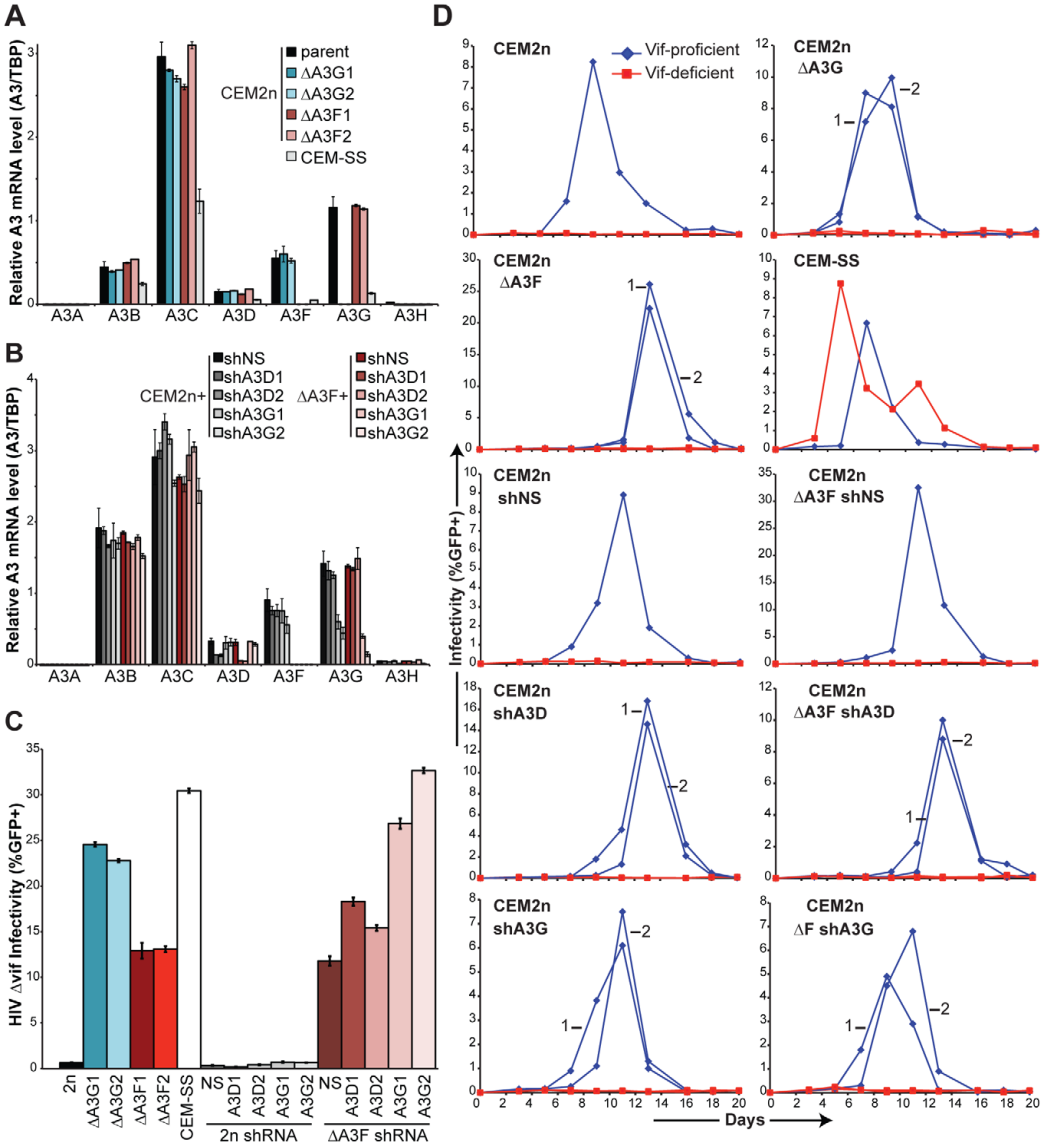
Characterization of independent knockout and knockdown clones. (A) Levels of each indicated *A3* mRNA in CEM2n, *A3G*- and *A3F*-null derivatives, and CEM-SS (relative to *TBP*; mean and s.d. shown for triplicate experiments). (B) Levels of each indicated *A3* mRNA in CEM2n or *A3F*-null cells transduced with shNS, shA3D, or shA3G constructs (relative to *TBP*; mean and s.d. shown for triplicate experiments). (C) Single-cycle infectivity of Vif-deficient HIV produced in parallel in the indicated cell lines (mean and s.d. shown for p24-normalized triplicate experiments). (D) The kinetics of Vif-proficient (blue diamonds) and Vif-deficient (red squares) HIV spreading infection in the indicated cell lines. Numbers distinguish independent clones.

An unavoidable consequence of gene targeting is the clonal nature of the procedure required to generate biallelic knockout derivatives of a parental cell line. Additionally, the previously described single-cycle assays on *A3G*-null and *A3F*-null cell lines were done several months apart with virus stocks produced at different times. To minimize these potential effects, two independently derived subclones of each of the knockout lines, *A3G*-null and *A3F*-null, as well as two independent subclones from the *A3D* and *A3G* shRNA knockdown pools were generated and assayed in parallel. Q-PCR and immunoblotting was used to confirm knockdown efficiencies, 60–85% for *A3D* and 60–70% for *A3G* (Figure 5A & B). These cell lines were infected with VSV-G pseudotyped Vif-deficient HIV to determine infectivity levels after approximately one round of replication (Figure 5C). As above, deletion of either *A3G* or *A3F* increased viral infectivity an average of 38- or 20-fold, respectively. Knockdown of either *A3D* or *A3G* in the CEM2n background had little effect. In contrast, knockdown of *A3D* or *A3G* in the *A3F*-null background resulted in additional increases in Vif-deficient virus infectivity, averaging 27- and 49-fold higher than the baseline level in fully restrictive CEM2n cells expressing non-specific shRNA.

To assess the impact of *A3* deletion and knockdown on HIV replication over time, a series of parallel spreading infections were initiated on the same panel of cell lines (Figure 5A & B). Cells were infected at a multiplicity of infection of 1% with either Vif-proficient or Vif-deficient HIV_IIIB_. Every 2–3 days supernatants were removed to infect the reporter line CEM-GFP to assay live virus and, in parallel, to measure p24 levels (Figure 5D and Figure S6). Vif-proficient virus replicated on every cell line tested with peaks of infection occurring on days 7 to 13. The precise reason(s) for this kinetic variation is unclear but it does not seem to correlate with *A3* genotype (compare with Figure S6). As expected, Vif-deficient HIV replication was fully restricted in CEM2n cells but peaked readily in CEM-SS. *A3G*-null and *A3F*-null cells failed to produce detectable levels of infectious Vif-deficient virus, as monitored by the CEM-GFP (live virus) reporter system (although modest increases in p24 levels were detected in cell-free supernatants). In addition, knockdown of *A3D* or *A3G* in either the CEM2n background, or somewhat surprisingly, in the *A3F*-null background was unable to render cells fully permissive for Vif-deficient virus replication. Taken together, even though strong infectivity recoveries were evident in single round infections of cells completely lacking *A3G* or *A3F* or cells lacking *A3F* plus knocked-down *A3D* or *A3G*, the remaining endogenous A3s were still sufficient to restrict the spread of Vif-deficient virus (*i.e.*, those A3s that were not manipulated by knockout or knockdown). These data combined to suggest that the simultaneous elimination of A3D, A3F, and A3G (and possibly also A3H) would ultimately be necessary to render CEM2n fully permissive for Vif-deficient HIV replication.

## Discussion

Null mutations are a gold standard of genetics as they enable a definitive assessment of a given gene's function by comparing the phenotype of the wildtype parental state with that of an isogenic null derivative. Here, we report the identification of a new T cell line, CEM2n, derived from the common parental line CCRF-CEM. CEM2n is near-diploid, expresses CD4 and CXCR4, supports Vif-proficient but not Vif-deficient HIV replication, expresses a complex *A3* gene repertoire (similar to primary CD4+ T lymphocytes; Figure S2 & Ref. [2]), and is amendable to genetic manipulation by multiple methods including RNAi and gene targeting. We defined the HIV-restrictive A3 repertoire in this cell line by constructing *A3G*- and *A3F*-null derivatives, systematically depleting each expressed *A3* with specific shRNA constructs, and performing a series of Vif-deficient HIV infectivity and proviral DNA hypermutation experiments. These data enable the conclusions that endogenous levels of A3D, A3F, and A3G combine to limit the infectivity of Vif-deficient HIV, that A3G acts alone to inflict 5′GG-to-AG hypermutations, and that A3D and A3F work together to elicit 5′GA-to-AA hypermutations. Since both 5′GG and 5′GA mutational patterns are common in patient-derived HIV proviral DNA sequences and a CEM2n-like *A3* repertoire is expressed in primary CD4+ T lymphocytes, we hypothesize that these three A3s will also be responsible for HIV restriction and hypermutation *in vivo* (*e.g.*, [10], [37], [38], [39], [40]).

However, despite considerable efforts from our group and others, A3H remains a debatable factor in HIV restriction. This is due partly to the fact that some A3H haplotypes are more stable at the protein level (haplotypes II, V, VII>>I, III, IV, VI [41], [42], [43]). Over-expression studies are mostly consistent showing that A3H-hapII can restrict Vif-deficient HIV, that Vif can counteract this activity, and that these interactions are conserved between the A3H orthologs of other species and their cognate lentiviral Vif proteins [20], [41], [43], [44], [45], [46]. A3H is also expressed in primary CD4+ lymphocytes and induced upon HIV infection [20], [44]. However, our present studies were not able to unambiguously address whether this enzyme contributes to HIV restriction because endogenous *A3H* mRNA levels in CEM2n are ∼20-fold lower than those in primary CD4+ lymphocytes [2] (Figure S2). Even so, we observed a significant increase in Vif-deficient HIV infectivity in *A3F*-null/*A3H*-knockdown cells in comparison to the *A3F*-null line suggesting that even sub-physiological levels of endogenous A3H may be restrictive. This observation is additionally interesting in light of sequencing data showing that CEM2n is homozygous for the haplotype II genotype.

Our present studies will also help resolve literature conflicts on the topic of Vif-deficient HIV restriction by A3A, A3B, and A3C (*e.g.*, A3A: [12], [14], [26], [47], [48], [49], [50]; A3B: [12], [19], [20], [51], [52]; A3C: [12], [14], [15], [51], [53], [54]). A3A is not expressed in CD4+ T lymphocytes or cell lines and is therefore unlikely to have a role [1], [2], [47], [55]. It is also Vif-resistant, suggesting that it is of little threat to the virus, and incapable of restricting Vif-deficient HIV even upon stable over-expression in permissive T cell lines (*e.g.*, [20]). A3B is expressed at low levels in CD4+ T lymphocytes and some T cell lines such as CEM, and its over-expression potently restricts HIV in the HEK293T model system [1], [2], [12], [20], [51], [52]. However, A3B is also insensitive to HIV Vif and does not restrict Vif-deficient HIV or inflict hypermutations when stably over-expressed in permissive T cell lines [12], [19], [20], [41], [51], [52], [56]. Taken together with data shown here that that *A3B* knockdown in parental CEM2n or the *A3F*-null background has no impact on virus infectivity or hypermutation profiles, we conclude that endogenous A3B also does not contribute to HIV restriction. A3C is highly expressed in primary CD4+ T cells and T cell lines, exhibits a 5′GA-to-AA hypermutation preference (especially with SIV-based viral substrates), and is susceptible to HIV Vif [2], [51], [54]. However, when over-expressed in permissive T cell lines SupT11 or CEM-SS, A3C does not encapsidate or restrict Vif-deficient HIV replication [20]. Moreover, since *A3C* knockdown in the parental CEM2n or the *A3F*-null background had no impact on virus infectivity in our present studies, we conclude that endogenous A3C is also unlikely to contribute to Vif-deficient HIV restriction.

As elaborated in the Introduction, many prior published reports have been susceptible to a major weakness by being dependent upon enforced cDNA over-expression. Here, we overcome this drawback by identifying a new genetic system for host factor studies and systematically deleting and/or depleting endogenous *A3* genes. Our studies demonstrate that A3G is the sole source of 5′GG-to-AG hypermutations and, formally, that it is a minor (at best) contributor to 5′GA-to-AA hypermutations. Our studies are also consistent with the phenotypes of the molecular clones engineered to be preferentially susceptible to A3F or A3G [57], [58]. However, our studies are additionally unique by providing an unanticipated demonstration of a major role for endogenous A3D in inflicting 5′GA-to-AA hypermutations. Thus, virtually all 5′GA-to-AA hypermutations can now be explained by overlapping A3D and A3F activities. Such an unambiguous assignment of function would not have been possible by simple over-expression studies because A3D and A3F have a similar 5′TC deamination preferences. Altogether, we conclude that endogenous levels of three enzymes – A3D, A3F, and A3G – combine to inflict signature G-to-A hypermutations and mediate HIV restriction.

## Materials and Methods

### Cell Lines

All T cell lines were maintained in RPMI supplemented with 10% fetal bovine serum (FBS) and 0.5% penicillin/streptomycin (P/S). Our original CEM (4n) line was obtained from Michael Malim [28]. The initial CCRF-CEM line used here was obtained from the ATCC (cat #CCL119), and it was subcloned by limiting dilution to obtain multiple daughter clones including CEM2n. The HIV infectivity indicator cell line CEM-GFP was obtained from the AIDS Research and Reference Reagent Program. HEK293T cells were cultured in DMEM supplemented with 10% FBS and 0.5% P/S.

### Karyotype Analyses

Giemsa banding and karyotype determination were performed at the University of Minnesota's Cytogenetics Laboratory.

### rAAV Targeting Constructs

Generation of rAAV targeting vectors was performed as described [59]. Homology arms were selected to avoid identity with other *A3* genes and repetitive sequences. CEM2n genomic DNA was prepared using Gentra Puregene Cell Kit (Qiagen) and 1 kb homology arms were amplified using high fidelity PCR (Platinum Taq HiFi, Invitrogen). Primer sequences and genomic location of arms are listed in Table S2. Arms were cloned into pJet1.2 using CloneJet PCR cloning kit (Fermentas) and sequence verified. Left arms were digested with *Spe*I and *Not*I and right arms were digested with *Sal*I and *Not*I. pSEPT [59] plasmid was digested with *Spe*I and *Sal*I and pAAV-MCS containing the viral ITR was digested using *Not*I. rAAV vectors were then constructed via four-way ligation and purified using standard phenol∶chloroform extraction followed by ethanol precipitation. 20 µl of each ligation mixture was used to transform 10 µl electro-competent DH10B cells. Plasmid DNA was harvested from ampicillin resistant colonies and verified by restriction digest and DNA sequencing.

### AAV Virus Production, Infection of Target Cells

AAV-2 viral stocks were prepared by co-transfecting HEK293T cells at 50% confluency with sequence verified rAAV targeting vector, pHelper and pAAV-RC (Stratagene) (Trans-IT, Mirus). Three days after transfection, media and cells were collected and subjected to 3 cycles of freeze-thaw-vortex (30 min at −80°C, 10 min thaw at 37°C, 30 s vortex). Cellular debris was removed by centrifuging for 30 min at 12,000 rpm in a table-top centrifuge. rAAV was further purified and concentrated using an AAV Purification ViraKit (Virapur) per manufacturer's instructions. One million CEM2n recipient cells were infected with a range of volumes (2–100 µl) of virus. Three days after infection, cells were seeded into 96-well plates at a density of 1000 cells/well in G418 containing media (1 mg/ml). G418 resistant clones were expanded and harvested for genomic DNA. Clones were screened for homologous recombination events at the desired locus with PCR primers specific to upstream (5′), downstream (3′) regions of the targeted allele, and the targeting vector. To recycle the targeting construct for the second allele, clones were infected with a Cre-expressing adenovirus (Ad-Cre-GFP, Vector Biolabs). Cells were cloned by limiting dilution and tested for drug sensitivity.

### RNA Isolation, cDNA Synthesis, and Q-PCR

mRNA isolation, reverse transcription and Q-PCR were performed as described [2]. RNA from 5×10^6^ cells was isolated using the RNeasy kit (Qiagen). 1 µg total RNA was used to synthesize cDNA with random hexameric primers (AMV RT; Roche). cDNA levels were quantified using established procedures, primers, and probes [2] using a Roche LightCycler 480. All reactions were done in triplicate and *A3* levels were normalized to the housekeeping gene *TBP* and presented relative to values for the parental CEM2n line.

### Viral Constructs

Vif-proficient (Genbank EU541617) and Vif-deficient (X26, X27) HIV_IIIB_ A200C proviral constructs have been described [18], [19].

### Spreading Infections

Vif-proficient and Vif-deficient HIV spreading infections were performed as previously described [18]. For p24 ELISA, anti-p24 mAb (183-H12-5C, NIH ARRRP) coated 96-well plates (maxisorp, Nunc) were incubated with supernatants from infected cultures for 1 hr. Following 3 washes with PBS 0.1% Tween 20 (PBS-T), a second 1 hr incubation with a different anti-p24 mAb (9725, N. Somia) was used to ‘sandwich’ the p24 antigen. After an additional 3 PBS-T wash steps, p24 was quantified by 0.5 hr incubation with an enzyme-linked secondary goat anti-mouse IgG-2A/HRP followed by 3 PBS-T wash steps and incubation with 3,3′,5,5′ tetramethybenzidine (TMB) for 6 min. The reaction was stopped upon addition of 1 M H_2_SO_4_ and absorbance at 450 nm was quantified on a microplate reader (Synergy MX, Biotek).

### Single Cycle Infections

HEK293T cells were co-transfected at 50% confluence with a Vif-deficient HIV proviral expression construct and a VSV-G expression construct. Viral containing supernatants were harvested, titered on CEM-GFP, and used to infect CEM2n and A3-null derivatives at a 25% initial infection. 12 h post-infection cells were washed to remove remaining VSV-G virus and resuspended in RPMI growth media. 36 h later, viral containing supernatants were used to infect CEM-GFP reporter cells and cell and viral particle lysates were prepared.

### Immunoblotting

Cell pellets were washed and directly lysed in 2.5× Laemmli sample buffer. Viral particles were isolated from filtered supernatants by centrifugation and then resuspended in 2.5× Laemmli sample buffer. SDS-PAGE and immobilization of protein to PVDF was carried out using the Criterion system (BioRad).

### Flow Cytometry

Immunostaining with CD4-PC7 and CXCR4-PE (Beckman Coulter) was carried out per the manufacturer's instructions. CEM and CEM2n stained negative for CCR5 (data not shown). Infected CEM-GFP cells were fixed with 4% paraformaldehyde in 1× PBS. Fluorescence was measured on a FACS Canto II instrument (BD), and data were analyzed with FlowJo Flow Cytometry Analysis Software (Version 8.8.6).

### shRNA Knockdown Vectors

pLKO.1 lentiviral vectors expressing short-hairpin RNA (shRNA) to A3B (TRCN00001420546), A3C (TRCN0000052102), A3D (TRCN0000154811), A3G (TRCN0000052191), and A3H (TRCN0000051799) were obtained from Open Biosystems. Lentivirus was produced by co-transfecting 50% confluent 293T cells with an shRNA expression construct, an HIV *gag-pol* helper plasmid, a VSV-G expression construct and Trans-IT (Mirus). Two days after transfection, supernatant was collected and replaced with fresh media. The following day, media was pooled and virus was clarified by passing supernatants through a 0.45 µM PVDF filter. To concentrate the virus, clarified supernatants were centrifuged at 22,000× g for 2 h. Virus containing pellets were resuspended in 1× PBS. One million CEM2n and *A3F*-null cells were infected with 50 µl of virus and two days post-infection, media was replaced with puromycin containing media (1 µg/ml). Subclones were generated by limiting dilution in 96-well plates. Knockdown efficiency and specificity on transduced pools and subclones was assessed with Q-PCR [2].

### 3D-PCR

3D-PCR was carried out as described [20]. A 511 bp amplicon from the *gag-pol* genes of integrated proviruses was amplified with degenerate primers and quantified (Roche, LightCycler 480). Normalized amounts of integrated provirus were used for a second round of PCR over a range of denaturation temperatures using a gradient thermocycler (Eppendorf).

### Mutational Spectra Analysis

48 h after infection of CEM-GFP cells with Vif-deficient HIV, genomic DNA was prepared and PCR was used to generate a 511 bp amplicon over the *gag-pol* region. Primer sequences are listed in Table S2. This amplicon was cloned into pJet1.2 using CloneJet PCR cloning kit (Fermentas) and sequenced. Duplicate sequences were discarded. Sequences were analyzed using Sequencher 4.6 (Gene Codes Corp).

## Supporting Information

Figure S1 **CEM is a near-tetraploid T cell line.** Giemsa-banding karyotype of a representative CEM metaphase spread. Red arrows indicate typical lesions in lymphoblastic leukemia.
(TIF)

Figure S2 **A comparison of** ***A3*** **mRNA levels in CEM2n, CEM(4n), and primary CD4+ lymphocytes.** Total RNA was produced from the indicated sources, 1 µg was converted to cDNA, and one-twentieth of each cDNA was used to prime the indicated Q-PCR reactions. Levels of *A3G* in CEM2n are set to 1 to facilitate comparison. See methods for additional details.
(TIF)

Figure S3 **PCR reactions to detect** ***A3G*** **targeting events.** (A) Schematic of a correctly targeted *A3G* locus. Diagnostic PCR reactions enable detection of the IRES-Neo cassette adjacent to the left targeting arm (PCR A = 1.8 kb) and adjacent to the right targeting arm (PCR B = 1.8 kb). An internal primer set enables detection of any drug resistant clone (PCR C = 1.9 kb). The flanking primers also enable detection of the *loxP*-to-*loxP* deletion product following Cre-mediated recombination (PCR D = 2.9 kb). (B) Agarose gel image of PCR products produced from the genomic DNA of CEM2n (WT), an *A3G* heterozygote, and an *A3G*-null clone.
(TIF)

Figure S4 **G-to-A mutation loads for Vif-deficient HIV produced in CEM2n and key derivatives.** The G-to-A mutation number per independent amplicon is shown in histogram format for Vif-deficient HIV recovered from single-round viral infections of the indicated cell lines. (A) CEM2n experiments 1 and 2 are combined into a single histogram. (B) *A3G*-null CEM2n. (C) *A3F*-null CEM2n. (D) *A3F*-null/*A3D*-knockdown CEM2n. The corresponding dinucleotide preferences are shown in pie format in Figures 2F, 3F, and 4E. The G-to-A mutations for all experimental conditions are summarized in Table 2 and the raw data can be found in Table S1. A trend toward higher G-to-A mutation loads and a bimodal mutation distribution was observed in viruses produced in parental CEM2n and in the *A3F*-null/*A3D*-knockdown derivative, suggesting that endogenous A3G may be more processive (not necessarily more anti-viral) than A3F or A3D.
(TIF)

Figure S5 **PCR reactions to detect** ***A3F*** **targeting events.** (A) Schematic of a correctly targeted *A3F* locus. Diagnostic PCR reactions enable detection of the IRES-Neo cassette adjacent to the left targeting arm (PCR A = 2.1 kb) and adjacent to the right targeting arm (PCR B = 1.7 kb). An internal primer set enables detection of any drug resistant clone (PCR C = 1.9 kb). The flanking primers also enable detection of the *loxP*-to-*loxP* deletion product following Cre-mediated recombination (PCR D = 2.8 kb). (B) Agarose gel image of PCR products produced from the genomic DNA of CEM2n (WT), an *A3F* heterozygote, and an *A3F*-null clone.
(TIF)

Figure S6 **Vif-Proficient and Vif-Deficient HIV replication kinetics.** Quantification of supernatant p24 levels at the indicated time points in (A) CEM2n and the indicated *A3F*- or *A3G*- null derivatives, (B) CEM2n subclones expressing the indicated shRNA constructs, and (C) CEM2n *A3F*-null subclones expressing the indicated shRNA constructs. The spreading infections in panels A, B, and C were done in parallel, and they are therefore directly comparable. Vif-proficient and Vif-deficient viral replication was done in parallel in CEM-SS (the only non-isogenic condition) and presented in panel A for comparison.
(TIF)

Table S1 **G-to-A hypermutation.** A full list of G-to-A hypermutations observed in Vif-deficient HIV-1 produced in CEM2n or the indicated derivatives. These raw data were used to create Table 2 and Figures 2F, 3F, 4E, and S6.
(XLS)

Table S2 **Primer sequences.** A full list of primer sequences used in this study.
(TIF)
