# Supplementary figures and images for: Endogenous Origins of HIV-1 G-to-A Hypermutation and Restriction in the Nonpermissive T Cell Line CEM2n
Source: PLoS Pathog. 2012 Jul 12;8(7):e1002800. doi: 10.1371/journal.ppat.1002800 (PMC3395617; doi:10.1371/journal.ppat.1002800)

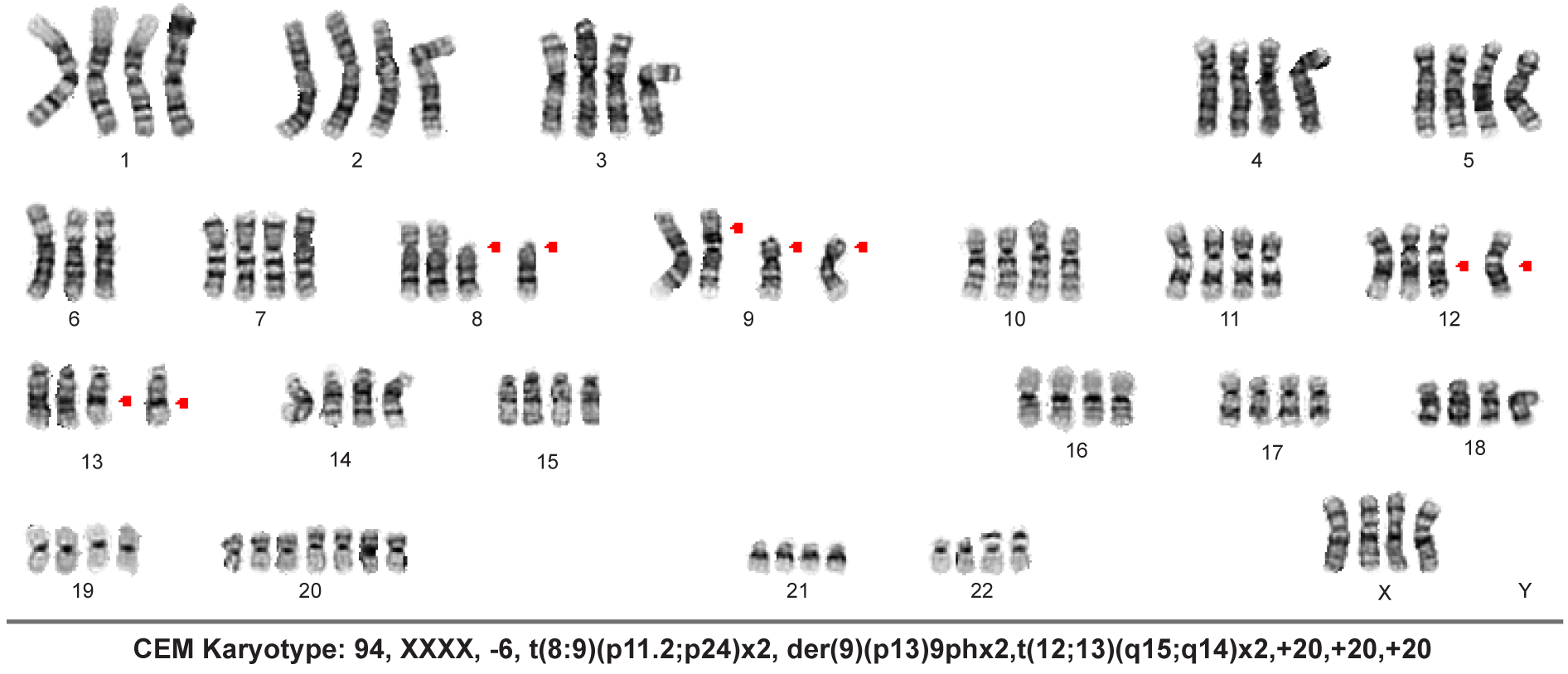

Supplement: Figure S1 — CEM is a near-tetraploid T cell line. Giemsa-banding karyotype of a representative CEM metaphase spread. Red arrows indicate typical lesions in lymphoblastic leukemia. (TIF) [file ppat.1002800.s001.tif]

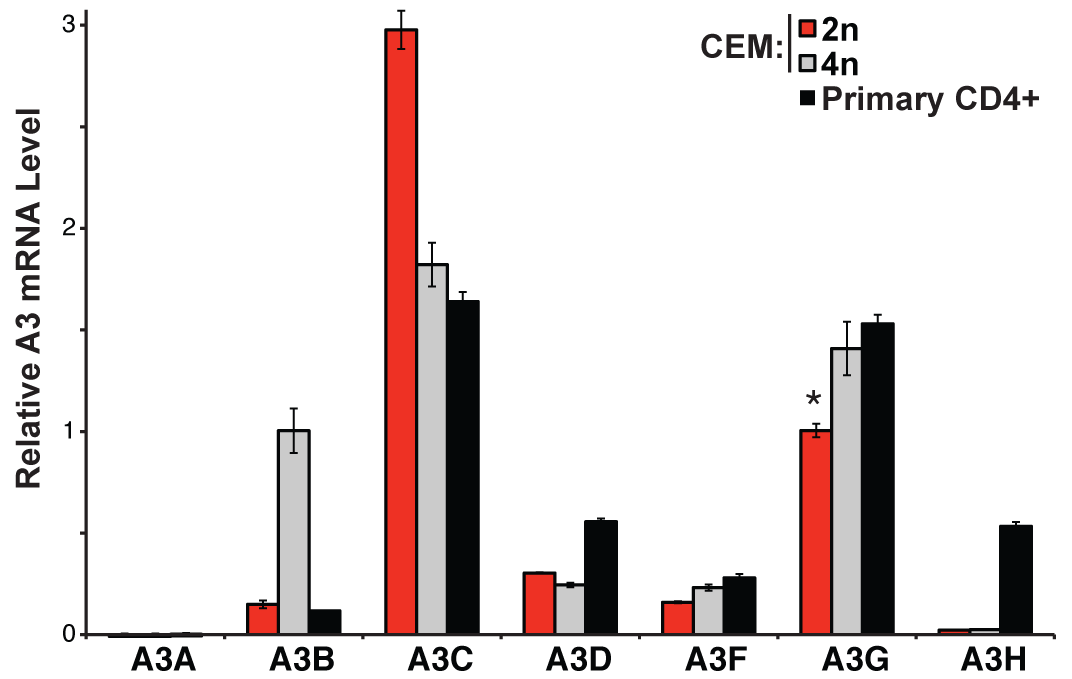

Supplement: Figure S2 — A comparison of A3 mRNA levels in CEM2n, CEM(4n), and primary CD4+ lymphocytes. Total RNA was produced from the indicated sources, 1 µg was converted to cDNA, and one-twentieth of each cDNA was used to prime the indicated Q-PCR reactions. Levels of A3G in CEM2n are set to 1 to facilitate comparison. See methods for additional details. (TIF) [file ppat.1002800.s002.tif]

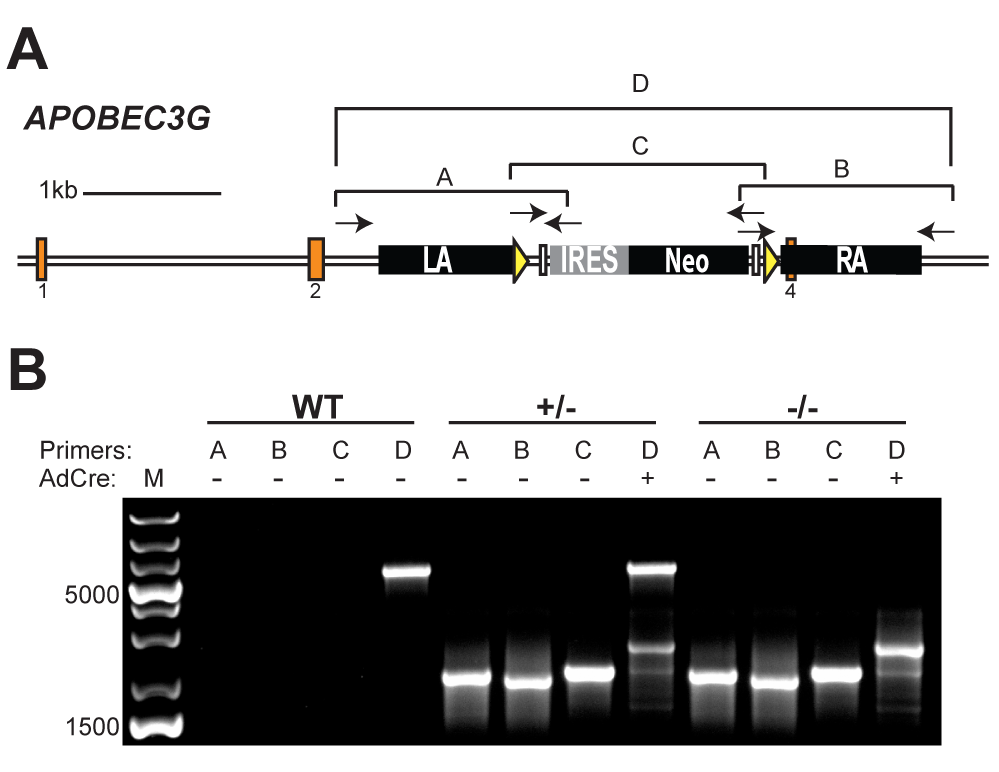

Supplement: Figure S3 — PCR reactions to detect A3G targeting events. (A) Schematic of a correctly targeted A3G locus. Diagnostic PCR reactions enable detection of the IRES-Neo cassette adjacent to the left targeting arm (PCR A = 1.8 kb) and adjacent to the right targeting arm (PCR B = 1.8 kb). An internal primer set enables detection of any drug resistant clone (PCR C = 1.9 kb). The flanking primers also enable detection of the loxP-to-loxP deletion product following Cre-mediated recombination (PCR D = 2.9 kb). (B) Agarose gel image of PCR products produced from the genomic DNA of CEM2n (WT), an A3G heterozygote, and an A3G-null clone. (TIF) [file ppat.1002800.s003.tif]

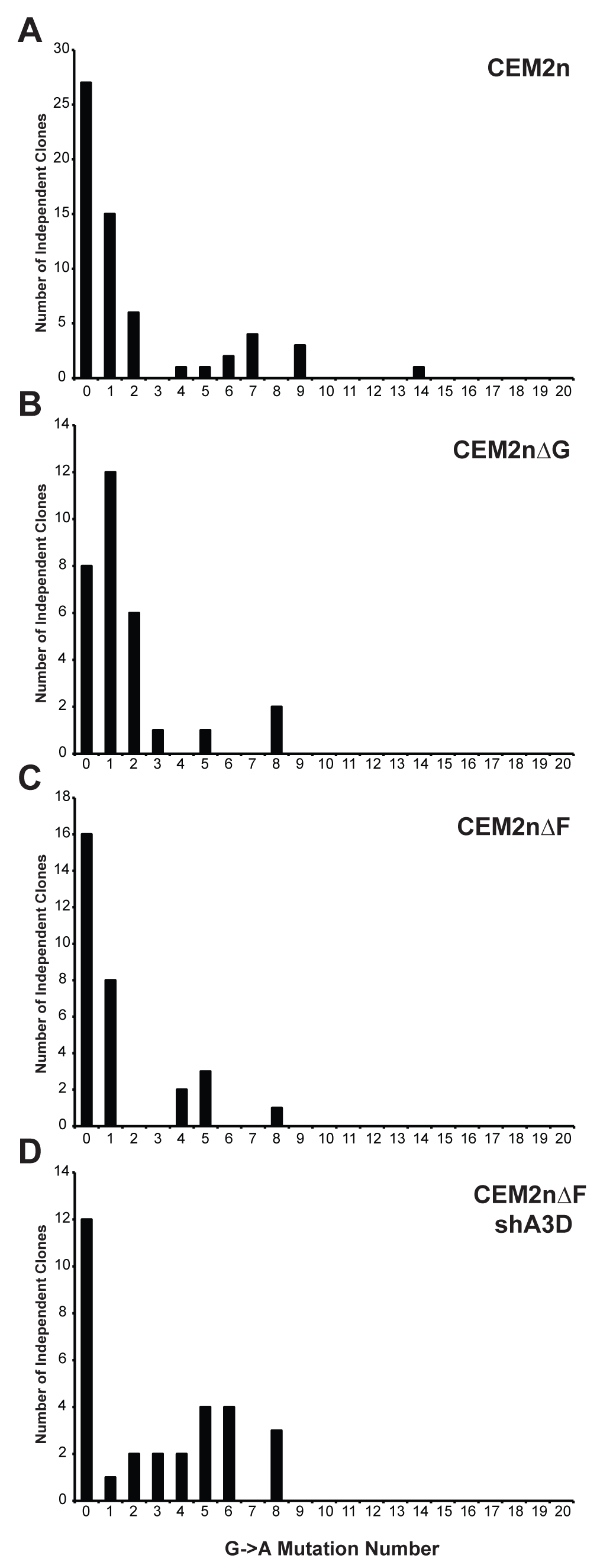

Supplement: Figure S4 — G-to-A mutation loads for Vif-deficient HIV produced in CEM2n and key derivatives. The G-to-A mutation number per independent amplicon is shown in histogram format for Vif-deficient HIV recovered from single-round viral infections of the indicated cell lines. (A) CEM2n experiments 1 and 2 are combined into a single histogram. (B) A3G-null CEM2n. (C) A3F-null CEM2n. (D) A3F-null/A3D-knockdown CEM2n. The corresponding dinucleotide preferences are shown in pie format in Figures 2F, 3F, and 4E. The G-to-A mutations for all experimental conditions are summarized in Table 2 and the raw data can be found in Table S1. A trend toward higher G-to-A mutation loads and a bimodal mutation distribution was observed in viruses produced in parental CEM2n and in the A3F-null/A3D-knockdown derivative, suggesting that endogenous A3G may be more processive (not necessarily more anti-viral) than A3F or A3D. (TIF) [file ppat.1002800.s004.tif]

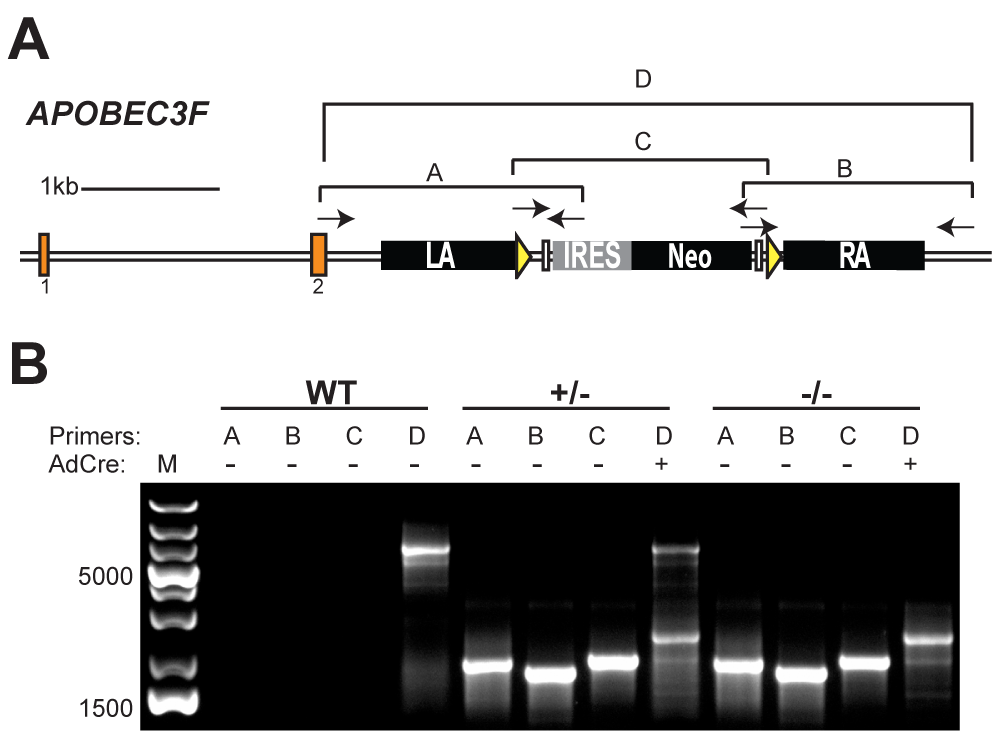

Supplement: Figure S5 — PCR reactions to detect A3F targeting events. (A) Schematic of a correctly targeted A3F locus. Diagnostic PCR reactions enable detection of the IRES-Neo cassette adjacent to the left targeting arm (PCR A = 2.1 kb) and adjacent to the right targeting arm (PCR B = 1.7 kb). An internal primer set enables detection of any drug resistant clone (PCR C = 1.9 kb). The flanking primers also enable detection of the loxP-to-loxP deletion product following Cre-mediated recombination (PCR D = 2.8 kb). (B) Agarose gel image of PCR products produced from the genomic DNA of CEM2n (WT), an A3F heterozygote, and an A3F-null clone. (TIF) [file ppat.1002800.s005.tif]

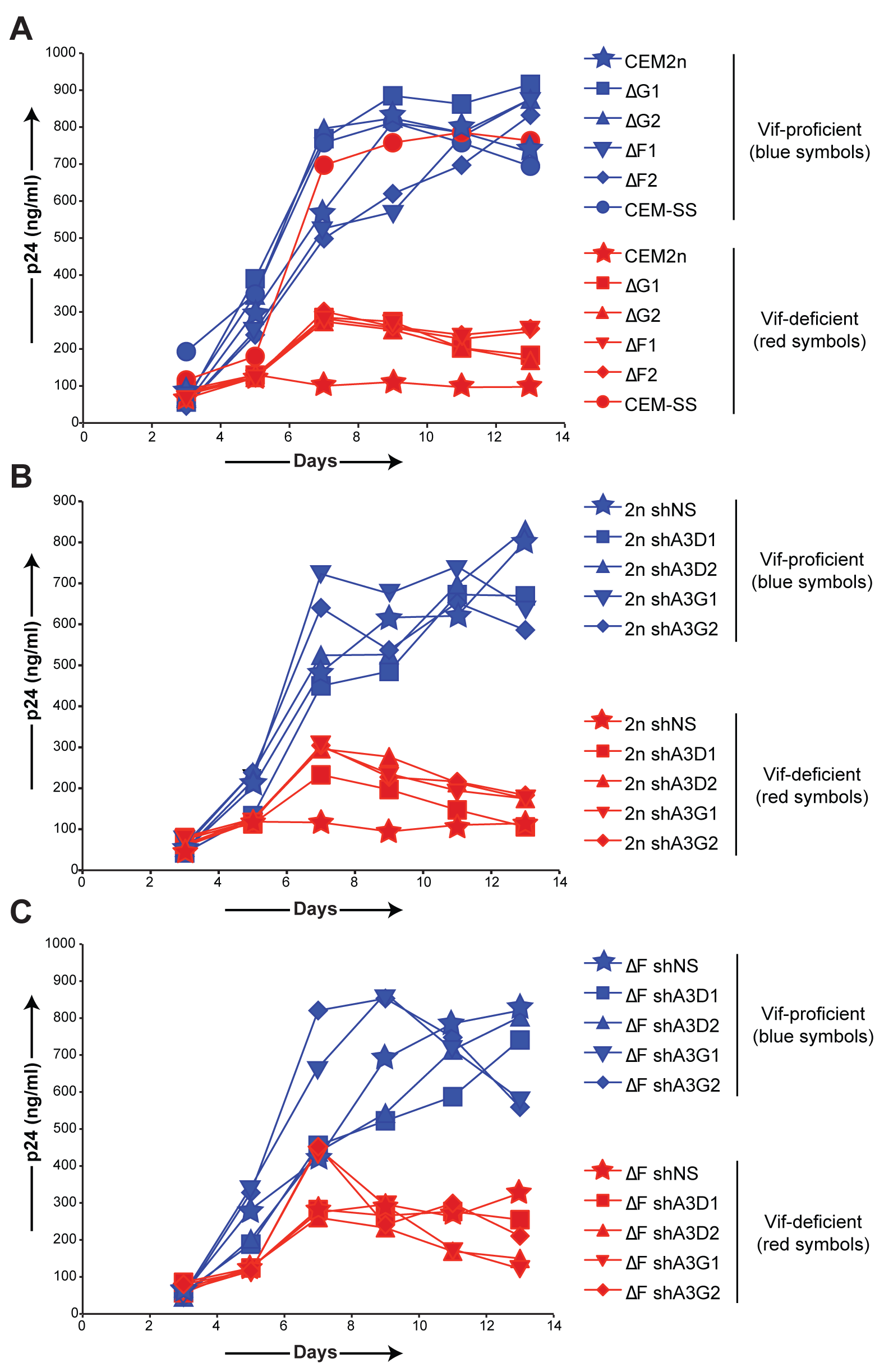

Supplement: Figure S6 — Vif-Proficient and Vif-Deficient HIV replication kinetics. Quantification of supernatant p24 levels at the indicated time points in (A) CEM2n and the indicated A3F- or A3G- null derivatives, (B) CEM2n subclones expressing the indicated shRNA constructs, and (C) CEM2n A3F-null subclones expressing the indicated shRNA constructs. The spreading infections in panels A, B, and C were done in parallel, and they are therefore directly comparable. Vif-proficient and Vif-deficient viral replication was done in parallel in CEM-SS (the only non-isogenic condition) and presented in panel A for comparison. (TIF) [file ppat.1002800.s006.tif]

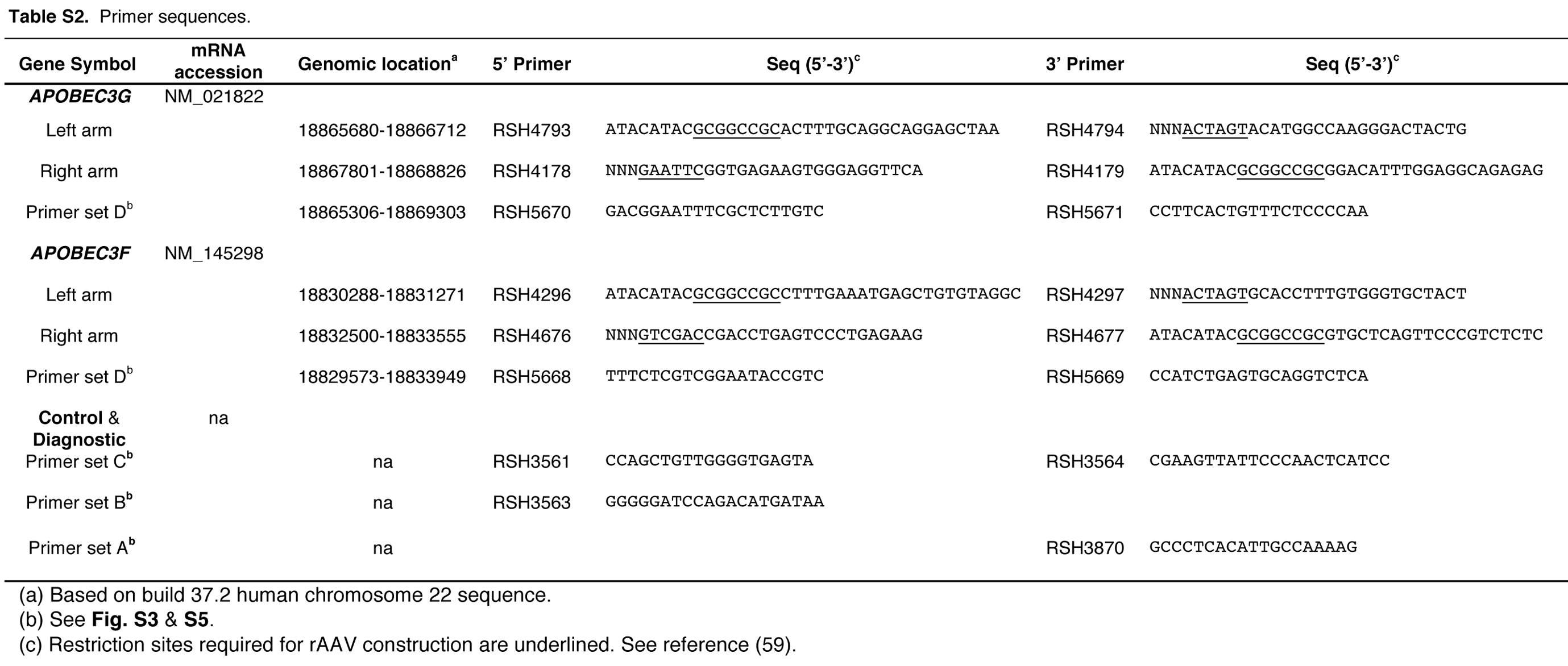

Supplement: Table S2 — Primer sequences. A full list of primer sequences used in this study. (TIF) [file ppat.1002800.s008.tif]
